# Proceedings of Northern Ohio Dental Society

**Published:** 1877-07

**Authors:** 


### PROCEEDINGS OF THE NORTHERN OHIO DENTAL SOCIETY.

*Eighteenth Annual Meeting of the Northern Ohio Dental Association, held at Cleveland, O., May 8th and 9th,* 1877.

The Association was called to order at 11 o'clock, A.M., by Prest. Siddall.

Reading of reports being in order; the Corresponding Secretary and Treasurer made their reports, which, on motion were adopted.

The Corresponding Secretary presented a bill for printing stationary, etc., and on motion an order was drawn on the Treasurer for the amount.

On motion the rule in regard to the reading of essays was suspended.

The Chairman of the Executive Committee then presented the following topics for discussion:

1st. Treatment of Alveolar Abscess.

2nd. What can be accomplished for the arrest of dental caries, either by local or general treatment, otherwise than by filling.

3rd. Extraction of the six-year molars; when indicated.

4th. Filling teeth; comparative value of different materials, and different methods of operating.

On motion the Association adjourned to meet at 2 o'clock, P.M.

The afternoon session commenced promptly at 2 o'clock, Prest. Siddall in the chair.

The first subject: Treatment of Alveolar Abscess was taken up.

Dr. L. Buffett opened the discussion with some very interesting and appropriate remarks and referred to the replanting of teeth for the cure of alveolar abscess.

Dr. J. E. Robinson. In my practice I have not found it necessary to remove a tooth for the purpose of curing alveolar abscess. It is true that many advocate this plan, but I have not yet considered it necessary and as a general rule think the same results can be secured by other means.

Dr. Lyder. We should be *very cautious* in this matter of replanting teeth. It is quite important that we select our patients with great caution. All conditions should be favorable. We should endeavor to understand the constitutional peculiarities of our patients and treat accordingly.

The Dr. then exhibited a large tumor, (the result of alveolar abscess) which he had removed; and gave his mode of treatment.

Further discussion of this subject was then postponed for the purpose of receiving candidates; whereupon Dr. Robinson proposed the name of Dr. J. C. Merritt, of Cleveland, Ohio, as a proper person to become a member of the Association.

A motion was then made and carried that Dr. Merritt be received into membership without examination as he was a member of the medical profession in good standing.

Dr. L. Buffett proposed the name of Dr. E. W. Pool as a suitable person to become a member of this Association—Dr.

Pool having passed a satisfactory examination before the Board was on motion received into membership.

Discussion of the subject resumed.

Dr. Peebles. I still adhere to the old-fashioned manner of treatment in curing alveolar abscess. Have not yet found a case where I thought it *absolutely* necessary to extract the tooth to cure the abscess. Have replanted teeth when knocked out by accident, but in no other case.

He then related a case which came under his treatment of a young lady about 15 years of age, who, by an accident, had broken off the central superior incisors close to the margin of the gums.

When he first saw this case, the face and upper lip was very much swollen; the root of the left superior incisor was in position —but that of the right incisor was out of position, and the process badly broken, this root was taken, the process adjusted and the root replaced. Prescribed as a wash, diluted tincture of myrrh and arnica. In about three months this case was so thoroughly cured and healthy as to warrant restoring the crowns with gold, using Osmond's screws for the purpose. This operation was done under the mallet. While building upon the root which was replanted, it did not seem to be more sensitive or unpleasant to the patient than the same operation upon the other. The case progressed favorably, and may now be regarded as a successful operation.

Dr. Siddall being called upon to relate the present condition, in the case of replanting the central superior incisors of a young man in Oberlin, Ohio, reported at the last meeting, and treated by himself and Dr. Knowlton, reported that the case was still doing well and he regarded it as a permanent success.

Dr. Jennings. How treat the cavity after the extraction of a tooth and before replanting?

Dr. L. Buffett. I would cleanse out the cavity with something which will prevent the blood from coagulating. I would use alcohol as this has no affinity for the blood—is an antiseptic and very cleansing.

Dr. Jennings. I would here state that Dr. McKellop's mode of treatment was to wash out the cavity thoroughly with Pond's

Extract of Hamamelis; Dr. McKellop has treated 40 or 50 cases and has not yet had a failure, and he thinks it is better to extract and replant a tooth for the purpose of curing alveolar abscess than to break down the process from the outside in order to effect a cure.

Dr. Gibbons. Those who advocate and practice the extraction and replanting of teeth for the purpose of curing alveolar abscess are great enthusiasts, and some allowance must be made for a little exaggeration. I think there are nine cases out of ten of failures in the matter of replanting teeth. Have known abscess to follow the filling of superficial cavities in teeth, where there was no exposure of the nerve. It will not do to say that there are no failures or that all cases of alveolar abscess can be cured by the extraction and replanting method.

Dr. Jennings. There may have been some failures in replanting teeth; but they are few; numerous well anthenticated cases are reported every year, of the permanent cure of alveolar abscess by extracting and replanting. Cases are reported by men of such high standing in the profession that we cannot and dare not question them. Where the treatment is not thorough and where the conditions are all unfavorable there may have been a few failures, but that there are any such number of failures as suggested by Dr. Gibbons I cannot believe.

Dr. Lyder. In this matter of replanting teeth we must proceed very cautiously, and exercise our best judgment.

After replanting we must not abandon the case, thinking we have done all that can be done. The case should be under our frequent observation, and we should understand, and use the very best therapeutical agents.

On motion this subject was passed and the second, viz. What can be accomplished for the arrest of dental caries, either by local or general treatment, otherwise than by filling?

Dr. Robinson. If I were asked by a patient what could be done for the arrest of decay in the teeth otherwise than by filling, I would say nothing unless the decay is *very* superficial.

Dr. Jennings. In some cases the decay may be filed or cut out, the surface polished and burnished, and in this way further decay may be arrested for a long time, but this is advisable only in very superficial decay. Cleanliness most certainly affords valuable aid in arresting decay.

Dr. Spilman. This subject is a very comprehensive one; it embraces many conditions.

Decay is the result of chemical action; changing or destroying some of the constituent elements of tooth-structure. When you have filed off the enamel, cut out the decay, and polished the surface, the decay will not then be arrested, unless the chemical action is corrected. We must strike at the agents which cause the decay of dental tissue. The mouth and teeth must be kept clean; the fluids of the mouth restored to normal condition, and we must not neglect to give our patients proper instruction in regard to this matter. An excess of either alkali or acid is injurious to the teeth.

Dr. Lyder. I think we are often remiss in our duty to our patients. We fill their teeth, put them in good condition, as we think, and then dismiss them without advising or instructing them as to future care of their teeth; and however delicate a matter it may seem to be, we should *urge* upon our patients the necessity of cultivating habits of cleanliness in regard to their teeth.

Dr. Stephan. When there is white superficial decay around the necks of teeth, what treatment is indicated?

Dr. Butler. In my opinion it is not the acid but rather the alkaline condition of the mouth which does so much mischief. This case called up by the enquiry of Dr. Stephan is but an expression of the condition of the system. There is indicated some general disturbance; and when this decay, referred to, is removed, it will return again if the general system remains in the same condition. The best way to treat such decay is to cut out and polish the surface, leaving the tooth in as smooth and normal condition as possible In a well polished surface and the enamel in its normal condition there is no perceptible difference.

Then when all has been done which could be done mechanically—the cause of this condition must be reached through the general system.

Dr. Spilman. When the enamel rods or tubuli are exposed or broken into, they cannot be polished so as to make them as good as when in their normal or unexposed condition.

There is an enamel cuticle over the enamel for its protection, and I would ask Dr. Butler if he can see the enamel rods as readily when the enamel is in its normal condition, as when the enamel is broken into, and the surface polished?

Dr. Butler. The enamel cuticle cannot be found on a tooth after a little use. It is only found on newly erupted teeth, or on teeth which have not been used. Whether the enamel has been worn by contact with other teeth in the mouth or cut down, and polished by instruments there can be no perceptible difference.

Dr. Spilman. I do not regard it as good practice to file away the enamel to arrest decay. The enamel rods should always be protected as much as possible. This white zone around the necks of the teeth referred to by Dr. Stephan, is the result of the decomposition of gelatine in the teeth; the acid condition of the mouth eating it out. Whenever we discover roughness about the enamel, there is indication of decay commencing, or of some disorganizing agents at work.

On motion the Association adjourned to meet at 8 o'clock P.M.

EVENING SESSION.

Meeting called to order at 8 o'clock, by President Siddall. Subject under discussion continued.

Dr. Spilman. We need to understand more of chemical action and pathelogical conditions, in order to be more successful in our efforts to arrest decay. Acids have adjuncts, and we should know where and what they are, in order to combat them. In order to prevent decay, we should prevent the formation of the acids, or of the conditions of the mouth, which accomplish the decay.

L. Buffett. This case referred to, of white decay around the necks of teeth, is doubtless the result of an excess of acid; and general treatment must be resorted to. This is one of the signs of a deranged condition of system. The food has not been sufficiently nutritious or has not been assimulated. Local treatment should be in the direction of correcting the abnormal condition of the acids of the mouth; and in this case, I would use prepared chalk.

Dr. Palmer. I am not worth much for telling the why and wherefore, but the how I do things. I am for practical work. In the case referred to I would rub, polish, and burnish down the surface and get it as much like the normal enamel as posible. It is true that it will be an artificial surface, but I am satisfied it is the best thing that can be done.

Dr. Whitslar. We quite often meet with such cases as this now under discussion; and it is sometimes quite a question to decide what is the best treatment. I have sometimes resorted to the use of nitrate of silver and in this way have held, in check, the decay. Constitutional treatment is very important. The forces of nature must be put in good condition in order to overcome this tendency to the decay of dental structure.

Dr. Horton. This subject, under consideration, is the result of many causes: climate, mode of living, etc., produce this condition. It is one manifestation of the malarious condition of the system and should be treated constitutionally.

Dr. Palmer being called upon made some very appropriate remarks, in which he referred to Prof. Arthur's method of treating a great many decayed teeth. Prof. Arthur was very nice and particular in all his operations. He would carefully cut out the decay, leave no sharp angles—and preserve the contour of each tooth as much as possible, then he would, with great care, polish and burnish the surface. This practice is now pretty generally condemned, and he considered it a misfortune, when he was obliged to cut away any considerable portion of the tooth. Would attempt to keep a tooth in good shape and form, by filling rather than shaping by cutting and filing merely.

Dr. Horton. My teeth were filed apart when quite young: When much decay exists, saparating the teeth will not arrest it; but in superficial decay it often proves very useful. We have to resort to all sorts of expedients to preserve the teeth. No rules can be laid down; each one must use his best judgment. I know of a case where the front teeth were separated with the file years ago and are still in quite good condition while other teeth not filed apart have perished by decay.

I always endeavor to preserve the contour of a tooth, but there are some cases where this cannot be done and then we must resort to other methods.

Dr Palmer. When the decay has penetrated through the enamel. I always fill, but when not entirely through the enamel I file or cut it out and carefully polish—I agree with Dr. Horton that in some constitutions the teeth will not decay when filed apart.

On motion the Association adjourned to meet at 10 o'clock next morning.

---

*Wednesday Morning, May 9th,* 1877.

Meeting called to order by Prest. Siddall. On motion the Association proceeded to the election of Officers for the ensuing year, the result of which was as follows:

For President, J. W. Lyder; for Vice President, J. Stephan; for Treasurer, J. E. Robinson; for Recording Secretary, J. F. Siddall; Corresponding Secretary, F. S. Whitslar; Board of Examiners, S. P. Hildreth, L. Buffett and F. S. Whitslar; Executive Committee, J. E. Robinson and L. Buffett.

A motion was made and carried that when the Association adjourns it shall adjourn to meet in Elyria, Lorin County, Ohio, on the second Tuesday in May 1878.

On motion Drs. L. Buffett, J. W. Lyder, and D. R. Jenning were appointed a committee to select delegates to the American Dental Association which meets on the first Tuesday in August 1877, at Chicago.

Dr. J. W. Lyder, Prest. elect, was escorted to the chair by Drs. Robinson and Peebles, and made a few appropriate remarks.

Dr. J. F. Siddall on retiring—spoke of the progress made in dentistry since the organization of this association; and related some pleasant incidents in his own practice.

On motion of Dr. Whitslar the third subject was passed and the fourth : filling teeth, etc., taken up.

Dr. Whitslar. I made the motion to take up the fourth subject for the purpose of hearing from Dr. Field of Detroit, Mich. The word comparative in the subject now before us indicates that some materials are better than others for filling teeth. As a general thing gold has been considered the best. But I think there are exceptions; in white, chalky teeth, tin often answers a better purpose than gold. Tin in such teeth sometimes produces some change or chemical action, which we cannot perhaps explain, that tends to harden them. No one, I trust, doubts that good fillings have been and are still made of tin. I have seen teeth preserved for years by tin fillings. In some cases, in the front teeth, when the enamel is thin and transparent, gold may be placed around the walls of the cavity and then the tooth filled with tin. This may be done not only with less liability to fracture the enamel but for the sake of appearance and economy. I regard all materials for filling teeth as useful in their proper place. Thoroughness is the main thing, whether, filling with gold, tin or amalgam.

Dr. Field. We are all apt to have our hobbies. I think tin makes just as good fillings as gold ; but we must discriminate. What will do in one case or in one class of teeth will not do in another. I use very little amalgam; but I do use it when I deem it necessary. The Sterling and Laurence's amalgams I consider good. I do not wash them but put them into the cavity dry as possible.

In regard to gold I would say that Leslie's crystal gold will stand the test for contour fillings better that any other, but it must be used with care and in the right way, must be used with serrated and not smooth pointed instruments.

Watt's sponge gold is apt to roll around the instrument and from the walls of the cavity : but I do not find it so in using Leslie's. I make use of the electrical mallet: it is preferable to any other and less painful to the patient.

Dr. Palmer. I sometimes use gold and tin in the same cavity. I place the gold about the walls of the cavity for appearance, and it is economical for those who cannot afford large gold fillings. I have not had any bad results through chemical action by filling in that way. I think experience contradicts the theory that there is galvanic action between gold and tin. I think tin often preserves teeth better than gold, and I consider it particularly useful in preserving the teeth of the young. Of late years amalgams have been greatly improved—and I am now more inclined to use them than formerly. In the use of gold one should accustom himself to a certain kind and stick to that. I regard the cohesive gold foils as the best.

Dr. Ambler. I am one of the champions of tin foil. I never use amalgam. Tin foil I regard as the best material for filling soft, white teeth. There is a peculiarity about tin which tends to harden such teeth. I build out contour fillings with tin the same as with gold. Tin fillings are less liable to fail around the margin of the cavities near the gums than gold. In teeth with dead pulps tin is the best filling.

Dr. Buffett. I think we nearly all agree that the different materials we use in filling teeth are useful in their proper places. With me, gold is first, tin next, and amalgam as the last resort. Tin adapts itself better to the walls of a cavity than amalgam. Oxide of tin has a preservative effect upon the tooth; and a tin filling will be tolerated in a sensitive tooth where no other metalic filling will. On the masticating surface of a tooth I sometimes use amalgam, but never at the free margin of the gums. In putting in amalgam fillings we should be careful not to leave any sharp angles or corners—and they should be kept clean.

Dr. Palmer. Tin is very useful in filling teeth for young children: for the operation can be done quickly; and we all know it is important to save the deciduous teeth until it is time to shed them. I prepare the tin foil for filling either by rolling or folding it. I introduce one end of the strip and then build in fold after fold : do not cut up into blocks or pellets.

There are different ways for preparing gold for the instrument; I prefer the cylinder forms. In using cohesive gold I fill about the walls first: in non-cohesive gold I aim to keep the centre of the filling in advance.

Dr. Stedman made some appropriate remarks in which he stated that in selecting materials for filling, we should have reference to their mechanical and chemical effect.

Dr. Jennings related a case where he used Hill's Stopping, under the margin of the gums and completed the filling with gold—and the operation has proven a sucessful one.

Dr. Palmer stated that he does not fill teeth without the use of the rubber dam. In some special cases he made his own clamps: and in making the clamps, he would get a thick sheet of lead, and make first such a pattern as he needed by bending and shaping it so as to fit nicely the tooth to be filled; by this he would be guided in making the clamp. He then described his manner of making, and tempering a clamp.

On motion the association adjourned to meet at 2 o'clock P. M.

Afternoon session called to order promptly at 2 o'clock by Prest. Lyder.

The Committee appointed toselect, delegates to attend next Annual Meeting of the American Dental Association, presented the following names. B. T. Spelman, W. P. Horton, C. H. Harron, F. S. Whitslar, E. J. Way, D. R. Jennings, John Stephan, L. C. Kelsey, and J. C. Merritt. On motion the report of the committee on delegates was adopted.

The third subject, viz: Extraction of the Six Year Molar, when indicated, was discussed by Drs. Spilman, Ambler, Palmer, Buffett and others. Adjourned.

